# Thermal reaction products and formation pathways of two monoterpenes under in situ thermal desorption conditions that mimic vaping coil temperatures

**DOI:** 10.1038/s41598-023-49174-2

**Published:** 2023-12-08

**Authors:** Jianjun Niu, Jiping Zhu

**Affiliations:** Exposure and Biomonitoring Division, Environmental Health Science and Research Bureau, Health Canada, Ottawa, Canada

**Keywords:** Environmental sciences, Chemistry

## Abstract

Vaping has become more popular and different brands and types of vaping devices have rapidly emerged. However, little is known about the potential health risks of human inhalation exposures to the volatile chemicals in the vapour, which includes both directly vaporised components of vaping liquid and their reaction products formed during vaping processes. This study investigated reaction products of two monoterpenes (α-pinene and terpinolene) that are used as flavouring agents in vaping liquids with a focus on the identification of reaction products and their formation pathways. The thermal desorption was conducted under an in situ condition that is in the range of heating coil temperature in vaping by thermally desorbing the chemicals at a temperature range of 100–300 °C. Additional clean air was introduced during the thermal desorption. 36 and 29 reaction products were identified from α-pinene and terpinolene, respectively, at a relative concentration of 0.01% and greater in the desorbed mixture. 3-Carene was the dominant reaction product of α-pinene, while reaction products of terpinolene was dominated by p-isopropenyltoluene. Several reaction pathways including ring opening, allylic oxidation, cyclo-etherification, Wagner–Meerwein rearrangement, epoxidation, cleavage and removal of partial structure, and dehydration were involved in the formation of various reaction products. These pathways and resulting relative concentrations of residual parent compound and reaction products were influenced by both temperature and amount of air present during thermal desorption. The study results demonstrate possible existence of reaction products from thermally labile chemicals like monoterpenes in vaping aerosols and can help inform policies regulating vaping devices and products to protect public health.

Monoterpenes are common volatile organic compounds. They are used as flavoring agents in e-liquid in vaping devices^1–3^. However, these chemicals are known to be thermal liable and photoreactive due to unsaturated double bonds in the structures. Health concerns about inhalation of monoterpenes during vaping have been raised in recent years^4–6^ as vaping becomes more popular^7–9^. In addition to the choice of nicotine or cannabinoids, vaping liquids also contain many other components including a variety of flavouring agents such as terpenes^1–3^ and carrier substances (e.g., propylene glycol (PG), glycerol (GLY), and medium-chain triglycerides)^10,11^.

Thermal degradation in vaping has been confirmed to generate more transformed chemical products leading to increased unpredictable toxicity^10,12^. For example, soluble components of e-cigarettes are linked to dose-dependent loss of lung endothelial barrier function^13^. Benzene, a well known carcinogen^14^, has been found to be the common thermal degradant from many components in vaping liquids upon vaporization^10,15–17^. Other inhalation hazards (irritants and carcinogens) can also be generated from the thermal degradation including furans and aldehydes from saccharides (a sweet flavour for appealing children in vaping liquid)^18^, formaldehyde and dihydroxyacetone from PG and GLY^10,15^, and methacrolein and methyl vinyl ketone from tetrahydrocannabinol^19^. Therefore, consumers of vaping products are exposed from not only the vaporised vaping liquid components but also numerous thermal reaction products (RPs) of these components.

Vaping is associated with a number of adverse health outcomes. Diacetyl, once used in many e-cigarette flavors, was reported to causing severe lung damage^20^ and has been called by American Lung Association to be removed from e-cigarette cartridges. Other flavouring aldehydes in vaping liquids can form aldehyde-propylene glycol acetals which can activate irritant receptors in vitro^21,22^. Vitamin E acetate, a condensing agent in vaping products, has been suggested to be a possible cause of the 2019 outbreak of e-cigarette or vaping product use-associated lung injury (EVALI)^12^ which led to thousands of vape-related illnesses and dozens of deaths in the US^23^ The American Medical Association has made calls for a ban on, and Washington State has already banned, vape products containing vitamin E acetate^24,25^.

Although inhalation toxicity of oxidized monoterpenes has not been fully understood yet, the hazardous effects of the oxidized monoterpenes are well documented^26^. Atmospheric oxidation reactions of monoterpenes such as α-pinene, β-pinene, carene, limonene and terpinolene using smog chambers in the presence of ozone, hydroxyl radical or NOx as oxidants were also reported^27–31^. Although the reaction conditions of these studies are not directly applicable to those in vaping, the results of these studies are useful to predict possible formation of RPs to understand the reaction pathways of monoterpenes under vaping conditions^32^.

Limited reports on oxidative thermal reaction studies of monoterpenes mostly focus on offline reactions involving collecting reaction products during thermal reaction or pyrolysis in the presence of air, followed by solvent extraction or thermal desorption of the reaction products for GC/MS analysis^2,33,34^. Monoterpenes including pinane^35^, α-pinene^2,33,36^, β-pinene^35,37^, myrcene^19^, camphene, carene and limonene^33^ were employed in these studies. The formation of the RPs was dependent on experiment conditions such as device configuration, temperature and oxidants (O_2_, O_3_, OH^・^, NO_3_^・^, etc.). Major RPs from different pyrolysis reactions of α-pinene and/or β-pinene were reported; they include ocimene, allo-ocimene and limonene^35,36,38^, verbenol and verbenone^39^, and xylenes, benzene and ethylbenzene^37^. Thermal reaction of myrcene, on the other hand, produces benzene, methacrolein and isoprene.^19^ Five oxidative reaction pathways of several other monoterpenes (camphene, Δ3-carene, limonene, and α-terpinene) were also reported^33^. Most of these studies, however, were carried out at a temperature of 300 °C or higher, sometimes up to 800 °C^2,35,37^.

In our previous study, we demonstrated thermal oxidative reactions of several monoterpenes in an in situ thermal desorption GC/MS system in a lower temperature range of 100–300 °C^21^. In that study, formation of several reaction products peaks were observed but no structures and possible formation pathways of these products were identified. In addition, both temperature and oxygen were suspected to influence the thermal reactions of tested monoterpenes but the study didn’t conduct a detailed investigation on the influence of air intake. In this study, we further investigated the effects of air intake and temperature on thermal reactions of two monoterpenes, α-pinene and terpinolene, using an in situ thermal desorption GC/MS system operated at a temperature range of 100–300 °C^21^ and addition of clean air (air intake) to mimic vaping coil temperature and air pathways in vaping. The objective of the current study is to identify oxidative thermal RPs of α-pinene and terpinolene under the conditions that are relevant to situations in vaping, and to propose formation pathways for them.

## Methods

### Materials and sample preparation

Two monoterpenes that are commonly used in vaping liquids, α-pinene (purity: ≥ 99.0%) and terpinolene (≥ 94.0%), were obtained from Sigma-Aldrich Canada (Oakville, Ontario, Canada). The thermal reactions were carried out by pipetting 1 or 2 µl of the diluted monoterpene (1:1000 and 1:2000 in hexane for α-pinene and terpinolene, respectively) onto the glass wool that was packed inside in the middle (about 2 cm long) of a thermal desorption (TD) tube. After the sample introduction, the tube was flushed with a small mount of nitrogen gas (99.999%) (50 ml/min for 2 min, to a total purge gas volume of 100 ml) to disperse the liquid. Prior to pipetting, the packed tube had been conditioned prior to the experiment at 350 °C for 4 h under pure nitrogen gas with a flow rate of 100 ml min^−1^.

### TD-GC/MS

A Gerstel thermal desorption unit (TDU) with a cool injection system (CIS) coupled with Agilent 8890/5977B gas chromatography/mass spectrometry (GC/MS) was used to simulate the conditions used in vaping (temperature and air intake). A DB-624 column (60 m long with 0.25 mm I.D., and 1.4 μm film thickness) was employed in GC/MS for separation. GC oven temperature program was performed by keeping at initial temperature 40 °C for 15 min, then one step ramping to 250 °C at a rate of 10 °C min^-1^ and holding for 10 min at 250 °C with a total run time of 46 min. Temperature for the transfer line connecting to MS was set at 250 °C. MS was operated in Electron Impact (EI) mode with an ion source temperature of 230 °C and a quadruple temperature of 150 °C. The signals were detected in full scan mode with a scan range of 15–350 amu and solvent delay of 15 min.

The TD system has a two-stage thermal desorption process^21^ to transfer the evaporated chemicals into the GC column for analysis. Helium (He, 99.999%) was used as both desorption gas and GC/MS carrier gas. In the first stage, the TDU was operated with a split ratio of 1:100. It was heated from starting temperature of 40–100 °C at a rate of 60 °C min^−1^ and held for 3 min at 100 °C, while CIS temperature was kept at  − 10 °C. In the second stage, CIS was heated at a rate of 12 °C s^−1^ to the targeted temperature (100 °C or 200 °C or 300 °C) and held at that temperature for 5 min with a desorption of 100 ml min^−1^. The majority of the gas was vented; only 1 ml min^−1^ of the flow passed through the GC column. An additional air gas line, controlled by a needle valve, was added to the TDU to provide different levels of oxygen during the thermal desorption process. The needle valve was set at fully closed (level-1), a quarter turn opening (level-2) and a half turn open (level-3). At level-2 and level-3, the oxygen content, judged by the peak intensity of m/z 32 in GC/MS, increased about 50% and 200%, respectively, as compared with level-1. To protect the GC/MS system, the needle valve was manually opened during CIS desorption period (8 min) and closed when CIS desorption was completed. The initial oven temperature (40 °C) was maintained for another 7 min while the carrier gas (He) flow flushed out remaining air from the system.

### Unknown analysis

Agilent Unknown Analysis program (version 10) was used to putatively identify unknown peaks of GC/MS chromatograms. Suggested structures were provided by matching score in the NIST mass spectrum library for all peaks at a relative concentration of 0.01% and greater. A matching score of greater than 800 was generally required for the positive identification of the peak. In some cases where multiple matches of greater than 800 are present, a combination of the highest matching scores and analyst’s knowledge was employed for structure identification. In a few cases where distinctive fragment ions exist in the mass spectrum, a matching score between 700 and 800 was also accepted, with the judgement of the analyst, for structure identification. The peak areas were determined automatically by the program.

## Results and discussion

Two monoterpenes, α-pinene and terpinolene, were selected for this study due to their common presence in vaping liquids^40–42^. For each monoterpene, six thermal desorption conditions were tested: three different air intake levels at 200 °C and three different temperatures at air intake level-1. Thermal desorption GC/MS chromatograms of each experiment are provided in “Supplementary Material”. Temperatures of 100 °C for the first stage thermal desorption in a thermal desorption unit (TDU) and 100–300 °C for the second stage thermal desorption in the cool injection system (CIS) were used to best reflect common coil temperatures ranges in vaping devices. For example, Chen et al. reported coil temperatures of 145–334 °C and 110–185 °C under wet-through-wick and full-wet conditions, respectively, for the total of 13 replaceable coil heads of a second generation e-cigarette^43^. Our previous results^21^ have shown that using relatively low temperature of 100 °C in a TDU minimizes the possible reactions of the monoterpenes during the first stage desorption while ensuring sufficient evaporation and transfer of the monoterpenes to CIS. The temperature range of 100–300 °C in the CIS allows us to study temperature effects on the thermal reactions of monoterpenes. This temperature range is in the lower end of the coil temperatures used in various vaping devices.

### RPs of α-pinene and terpinolene

Reaction products (RPs) were putatively identified in the GC/MS chromatograms based on their match scores to the spectra in NIST library. Assigned identity of peaks and their percentages of total peak areas at three air intake (oxygen content) levels and three CIS temperatures from α-pinene and terpinolene are shown in Tables 1 and 2, respectively. For each identified chemical (column 2), its molecular formula, Chemical Abstracts Service (CAS) number, distinctive fragment ions (m/z) in the mass spectrum and its matching score with spectrum in NIST library are provided in column 3 through 6. A compound number is assigned to each chemical in the first column of the tables; these numbers will be used onwards in the paper to aid the discussion.

**Table 1:** Identified reaction products of α-pinene that have a percent area of 0.01% or greater.

Compound number^a^	Compound name	Formula	CAS number	NIST matching score	Distinctive fragment ions (m/z)	Air intake level (CIS 200 °C)			CIS temperature (^o^C) (Air intake level-1)		
						Level-1	Level-2	Level-3	100	200	300
1	α-Pinene	C_10_H_16_	80-56-8	979	136.1 93.1 121.1	50.01	44.84	42.73	56.94	49.73	41.65
**2**	3-Carene	C_10_H_16_	13466-78-9	974	136.1 93.1 121.1	26.39	25.33	24.67	22.50	26.71	23.76
**3**	24(10)-Thujadiene	C_10_H_14_	36262-09-6	968	134.1 91.1 119.0	6.16	6.83	9.27	3.52	6.09	9.38
**4**	α-Campholenal	C_10_H_16_O	4501-58-0	959	137.1 108.1 93.1	5.06	7.31	3.16	4.57	5.04	7.46
**5**	Limonene	C_10_H_16_	5989-27-5	986	136.1 68.0 93.0	4.18	3.01	3.39	1.77	4.10	3.92
**6**	p-Cymene	C_10_H_14_	99-87-6	974	134.0 119.0 91.0	1.62	1.70	1.72	1.23	1.60	2.32
**7**	Myrtenal	C_10_H_14_O	564-94-3	907	150.1 79.0 135.0	1.10	0.93	0.86	0.89	1.09	2.20
**8**	158-p-Menthatriene	C_10_H_14_	21195-59-5	942	134.1 91.0 119.0	0.69	0.93	0.90	0.61	0.71	1.19
**9**	o-Isopropenyltoluene	C_10_H_12_	7399-49-7	966	132.0 117.0 91.0	0.60	0.54	0.52	0.41	0.61	0.81
**10**	p-Isopropenyltoluene	C_10_H_12_	1195-32-0	921	132.1 117.0 91.0	0.49	0.50	0.46	0.18	0.49	0.98
**11**	o-Cymene	C_10_H_14_	527-84-4	973	134.1 119.0 91.0	0.45	0.39	0.42	0.31	0.45	0.56
**12**	177-Trimethylbicyclo[2.2.1]hept-5-en-2-ol	C_10_H_16_O	91055-72-0	929	137.0 108.0 93.0	0.41	0.54	0.33	0.41	0.41	0.72
13	Terpinolene	C_10_H_16_	586-62-9	969	136.1 121.1 93.1	0.40	0.28	0.26	0.25	0.39	0.26
14	Cosmene	C_10_H_14_	460-01-5	938	134.1 91.0 119.0	0.25	0.27	0.30	0.15	0.25	0.43
15	Camphene	C_10_H_16_	79-92-5	981	136.1 93.0 121.0	0.19	0.12	0.13	0.03	0.19	0.15
16	Pinocamphone	C_10_H_16_O	547-60-4	934	152.1 83.0 55.0	0.18	0.66	0.09	0.28	0.18	0.61
17	α-Phellandrene	C_10_H_16_	99-83-2	944	136.1 93.0 77.0	0.15	0.10	0.11	0.07	0.14	0.18
18	Eucalyptol	C_10_H_18_O	470-82-6	971	154.1 81.0 108.0	0.14	0.13	0.14	0.13	0.14	0.15
19	α-Terpinene	C_10_H_16_	99-86-5	926	136.1 121.0 93.0	0.13	0.09	0.10	0.06	0.13	0.08
20	Pinocarvone	C_10_H_14_O	30460-92-5	880	150.1 81.0 53.0	0.12	0.99	0.05	0.36	0.12	0.78
21	γ-Terpinene	C_10_H_16_	99-85-4	941	136.1 93.0 121.0	0.12	0.08	0.09	0.04	0.12	0.06
22	377-Trimethylcyclohepta-135-triene	C_10_H_14_	3479-89-8	925	134.0 119.0 91.0	0.05	0.05	0.05	0.04	0.05	0.07
23	Sabinene	C_10_H_16_	3387-41-5	915	136.0 93.0 41.0	0.05	0.04	0.03	0.01	0.05	0.05
24	β-Phellandrene	C_10_H_16_	555-10-2	894	136.0 93.0 77.0	0.04	0.02	0.03	0.02	0.04	0.03
25	Limona ketone	C_9_H_14_O	01-09-6090	823	138.1 95.0 67.0	0.03	0.05	0.04	0.05	0.04	0.06
26	Levoverbenone	C_10_H_14_O	1196-01-6	874	150.0 107.0 91.0	0.03	0.03	0.02	0.03	0.03	0.04
27	138-p-Menthatriene	C_10_H_14_	18368-95-1	893	134.0 119.0 91.0	0.03	0.03	0.04	0.01	0.03	0.04
28	Toluene	C_7_H_8_	108-88-3	931	91.0 65.0	0.02	0.04	0.05	0.01	0.02	0.05
29	3-Caren-10-al	C_10_H_14_O	14595-13-2	864	135.0 79.0 107.0	0.02	0.39	0.02	0.06	0.02	0.25
30	2-Caren-10-al	C_10_H_14_O	247911-68-8	781	150.1 107.0 79.0	0.02	0.02	0.02	0.01	0.02	0.03
31	3-Caren-2-ol	C_10_H_16_O	93905-79-4	894	134.0 119.0 91.0	0.02	0.02	0.02	0.01	0.02	0.04
32	2-Carene	C_10_H_16_	554-61-0	838	136.1 121.0 93.0	0.02	0.01	0.01	0.01	0.02	0.01
33	Cyclofenchene	C_10_H_16_	488-97-1	824	136.0 93.0 79.0	0.01	0.02	0.02	0.04	0.01	0.02
34	Bornylene	C_10_H_16_	464-17-5	800	136.0 93.0 121.0	0.01	0.02	0.02	0.01	0.01	0.02
35	Pinocarveol	C_10_H_16_O	547-61-5	853	134.0 92.0 55.0	0.01	0.08	0.01	0.03	0.01	0.08
36	Thujone	C_10_H_16_O	1125-12-8	756	152.0 110.0 81.0	0.01	0.03	0.01	0.01	0.01	0.02
37	m-Cymene	C_10_H_12_	1124-20-5	840	132.0 117.0 91.0	< 0.01	< 0.01	< 0.01	< 0.01	< 0.01	0.14
other	ΣUnknown					0.79	3.61	9.89	4.97	0.96	1.40

^a^ Bold number represents a major reaction product (RP); ∑Unknown: Total unknown product peaks.

**Table 2:** Identified reaction products of terpinolene that have percent area of 0.01% or greater.

Compound number^a^	Compound name	Formula	CAS number	NIST matching score	Distinctive fragment ions (m/z)	Air intake level (CIS 200 °C)			CIS temperature (^o^C) (Air intake level-1)		
						Level-1	Level-2	Level-3	100	200	300
13	Terpinolene	C_10_H_16_	586-62-9	963	136.1 121.1 93.1	34.64	34.42	34.55	40.76	34.61	31.32
**10**	p-Isopropenyltoluene	C_10_H_12_	1195-32-0	932	132.1 117.0 91.0	41.79	44.51	42.77	37.77	41.81	40.92
**38**	p-Cresol	C_7_H_8_O	106-44-5	821	107.0 77.0 51.0	4.39	4.74	4.70	5.10	4.38	2.99
**6**	p-Cymene	C_10_H_14_	99-87-6	965	134.0 119.0 91.0	3.96	5.75	6.39	3.93	3.95	5.91
**39**	Acetophenone	C_8_H_8_O	98-86-2	925	120.0 105.0 77.0	1.66	0.23	< 0.01	2.15	1.63	1.83
**40**	p-Cymen-8-ol	C_10_H_14_O	1197-01-9	944	150.1 135.0 91.0	1.52	0.21	0.10	1.33	1.53	0.28
**41**	Myrtenol	C_10_H_16_O	515-00-4	758	152.1 79.0 91.0	1.50	< 0.01	< 0.01	< 0.01	1.52	0.07
**42**	1-Methoxycyclohexa-14-diene	C_7_H_10_O	2886-59-1	780	110.0 79.0 95.0	< 0.01	0.04	0.05	< 0.01	< 0.01	< 0.01
**14**	Cosmene	C_10_H_14_O	460-01-5	904	134.1 91.0 119.0	1.48	< 0.01	< 0.01	0.52	1.48	2.86
**43**	14-p-Menthadien-7-al	C_10_H_14_O	22580-90-1	801	107.0 79.0 91.0	< 0.01	1.37	2.10	< 0.01	< 0.01	< 0.01
**27**	138-p-Menthatriene	C_10_H_14_	18368-95-1	943	134.0 119.0 91.0	1.10	1.26	1.78	0.51	1.08	2.37
**44**	Fenchol	C_10_H_18_O	1632-73-1	955	139.0 81.0 121.0	1.06	0.90	0.74	0.67	1.12	1.17
**8**	158-p-Menthatriene	C_10_H_14_	21195-59-5	936	134.1 91.0 119.0	0.92	1.05	0.97	0.89	0.94	0.96
**45**	Fenchone	C_10_H_16_O	1195-79-5	947	152.1 81.0 69.0	0.71	0.79	0.51	0.49	0.72	1.34
**32**	2-Carene	C_10_H_16_	554-61-0	936	136.1 121.0 93.0	0.67	0.77	0.80	0.60	0.65	0.61
**46**	2-Caren-4-ol	C_10_H_16_O	6617-35-2	868	152.1 109.1 137.0	0.52	0.79	0.40	0.08	0.50	0.49
**30**	2-Caren-10-al	C_10_H_14_O	247911-68-8	880	150.1 107.0 79.0	0.45	0.60	0.52	0.45	0.44	0.34
21	γ-Terpinene	C_10_H_16_	99-85-4	845	136.1 93.0 121.0	0.19	0.29	0.36	0.15	0.21	0.19
47	45-Epoxy-carane	C_10_H_16_O	6909-20-2	766	152.1 95.0 81.0	0.18	0.12	< 0.01	< 0.01	0.17	0.11
48	2-Methyl-5-(1-methylethenyl)-2-cyclohexane-1-ol	C10H16O	99-48-9	772	150.0 84.0 109.0	0.14	0.09	0.06	<0.01	0.14	0.12
19	α-Terpinene	C_10_H_16_	99-86-5	852	136.1 121.0 93.0	0.13	0.22	0.17	0.05	0.13	0.14
49	p-Acetyltoluene	C_9_H_10_O	122-00-9	840	134.0 119.0 91.0	0.12	0.10	0.07	< 0.01	0.11	0.17
22	377-Trimethylcyclohepta- 135-triene	C_10_H_14_	3479-89-8	771	134.0 119.0 91.0	0.10	0.04	0.03	< 0.01	0.09	0.17
5	D-Limonene	C_10_H_16_	5989-27-5	911	136.1 68.0 93.0	0.10	0.16	0.11	0.06	0.10	0.15
18	Eucalyptol	C_10_H_18_O	470-82-6	932	154.1 81.0 108.0	0.09	0.13	0.18	0.20	0.11	0.11
50	1-Methyl-14-cyclohexadiene	C_7_H_10_	4313-57-9	849	94.0 79.0 39.0	0.08	0.18	0.16	0.22	0.09	0.10
51	14-Cineole	C_10_H_18_O	470-67-7	850	154.1 111.0 125.0	0.08	0.14	0.16	0.08	0.09	0.14
52	cis-Dihydrocarvone	C_10_H_16_O	3792-53-8	760	152.0 67.0 109.0	0.08	0.06	< 0.01	< 0.01	0.06	0.14
53	p-Mentha-38-diene	C_10_H_16_	586-67-4	848	136.0 79.0 121.0	0.06	0.08	0.07	0.19	0.07	0.08
54	Benzaldehyde	C_7_H_6_O	100-52-7	916	106.0 77.0 51.0	0.05	0.08	0.05	< 0.01	0.06	0.09
other	ΣUnknown					2.24	0.88	2.21	3.80	2.22	4.81

^a^ Bold number represents a major reaction product (RP);  ∑Unknown: Total unknown product peaks.

**Figure 1:**
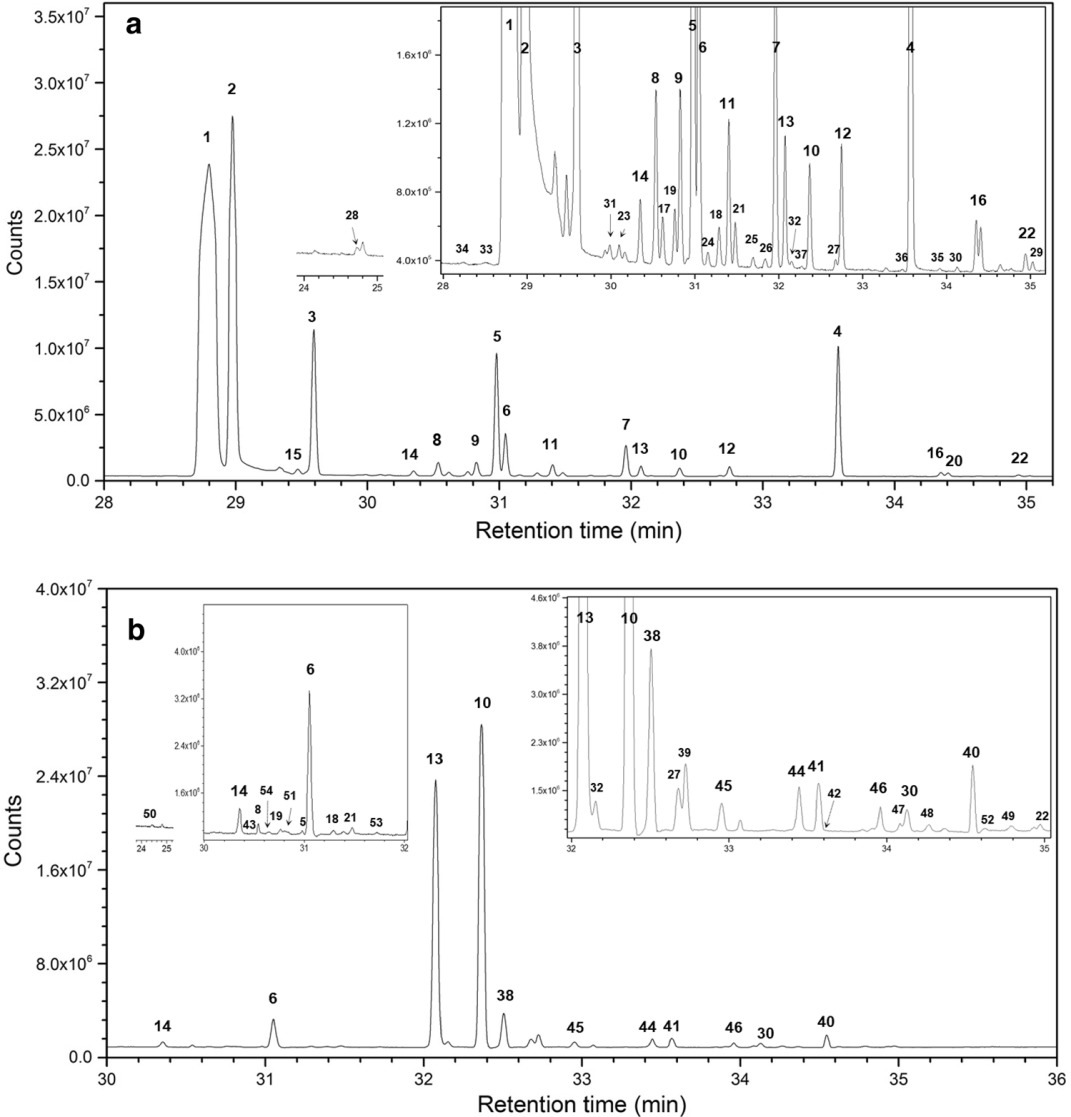
Thermal desorption GC/MS chromatogram at CIS temperature of 200 °C and air intake level-1. (**a**): α-pinene (**1**); (**b**) (terpinolene (**13**). Inserts in the chromatograms are enlarged sections to better show the small peaks.

Columns 7–9 in Tables 1 and 2 are results under varying air intake levels at the constant CIS temperature of 200 °C, while columns 10–12 are results under varying CIS temperatures at the constant air intake of level-1 (see Methods section for the definition of intake levels). Therefore, the experimental conditions for the results of column 7 and column 11 are the same; both are conducted at 200 °C and level-1 air intake. In both tables, the values in column 7 and column 11 were very similar, indicating good reproducibility of the experimental data. A representative chromatogram (level-1 air intake and CIS temperature of 200 °C) from α-pinene and terpinolene is shown in Fig. 1a and 1b, respectively.

In this study, due to lack of available standards for the majority of identified RPs, peak areas were used to estimate the relative concentrations of each peak. A relative concentration is the peak area of the compound divided by the sum of peak area of all the peaks multiplied by 100. A total of 36 RPs from α-pinene (**1**, for easy reference a number is assigned to each compound in Tables 1 and 2) were identified (Table 1), of which 11 PRs were detected at 0.5% and greater and considered major RPs. For terpinolene (**13**), the number was 29 and 16, respectively (Table 2). These 11 and 16 major RPs are indicated in the column 1 of the Tables 1 and 2, respectively. Not all peaks greater than 0.01% were positively identified; the sum of percent concentrations of these peaks (∑unknowns) is included in Tables 1 and 2 as well. The unknowns include peaks that could not be identified due to low matching scores (< 800) with NIST mass spectrum library or peaks with a relative concentration less than 0.01%. The sum of peak areas from unknowns represents a small portion (< 5%) of all peak areas in the study, except for the results of α-pinene (**1**) at air intake-level 3 (close to 10%, Table 1), suggesting more reactivity at higher air intake levels.

RPs from α-pinene (**1**) all have C10 structures, except for two minor RPs (limona ketone (**25**, C_9_H_14_O) and toluene (**28**, C_7_H_8_)) (Table 1). In the case of terpinolene (**13**), several RPs with fewer carbon atoms in their structures were observed. Most noticeable is the production of relatively high levels of p-cresol (**38**, C_7_H_8_O) and acetophenone (**39**, C_8_H_8_O). Other RPs of terpinolene (**13**) with fewer carbons included 1-methoxycyclohexa-1,4-diene (**42**, C7), p-acetyltoluene (**49**, C9), 1-methyl-1,4-cyclohexadiene (**50**, C7) and benzaldehyde (**54**, C7) (Table 2).

3-carene (**2**) was the most abundant RP from α-pinene (**1**), accounting for about 25% of all peak areas. It was followed by 2,4(10)-thujadiene (**3**), α-campholenal (**4**), limonene (**5**) and p-cymene (**6**). RPs **4** and **5** were also reported by heating α-pinene in the presence of air at a comparable temperature range of 90–130 °C^33^. RPs **2** and **6** have been reported, but not as major RPs of α-pinene, in smoke chamber-based atmospheric oxidation studies^11,44^. Thermal oxidative reactions of α-pinene have also been reported by others^2,35,36,38,39^, though the reaction conditions are different from those used in this study. Most of the reported studies were conducted by gas-phase pyrolysis, under which several RPs including myrtenal (**7**), pinocarvone (**20**), levoverbenone (**26**) and pinocarveol (**35**) were generated at 90 °C^39^ and **5** at around 250 °C^35,36^. Other RPs such as **6**, o-cymene (**11**), pinocamphone (**16**), pinocarvone (**20**), **26**, toluene (**28**) and **35** were reported^2^ from pyrolysis at higher temperatures of 300–800 °C, while **5**, terpinolene (**13**), α-terpinene (**19**) and γ-Terpinene (**21**) were also detected in the catalytic oxidation of **1**^38^.

p-Isopropenyltoluene (**10**) was the most abundant RP from terpinolene (**13**), accounting for about 40% of all peak areas. It is followed by p-cresol (**38**), p-cymene (**6**) and acetophenone (**39**). There is no report on the formation of these major RPs from terpinolene under thermal oxidative reactions. However, two of these major RPs (**6** and **10**) were detected upon the direct oxidation of terpinolene with oxygen in aqueous dispersion^45^. It is also interesting to note that a number of RPs (**5**, **6**, **8**, **10**, **14**, **18**, **19**, **21**, **22**, **27**, **30** and **32**, see Table 1 for chemical names) were detected from both α-pinene (**1**) and terpinolene (**13**) due to the transformation reaction of α-pinene to the same monocyclic terpene backbone of terpinolene via one of the reaction pathways of oxidative cleavage of the 6,7-carbon bond. Similarly, several of the same oxidative products were also found from pyrolyzing different monoterpenes with O_2_ in a sealed tube^33^.

### Reaction pathways of α-pinene

**Figure 2:**
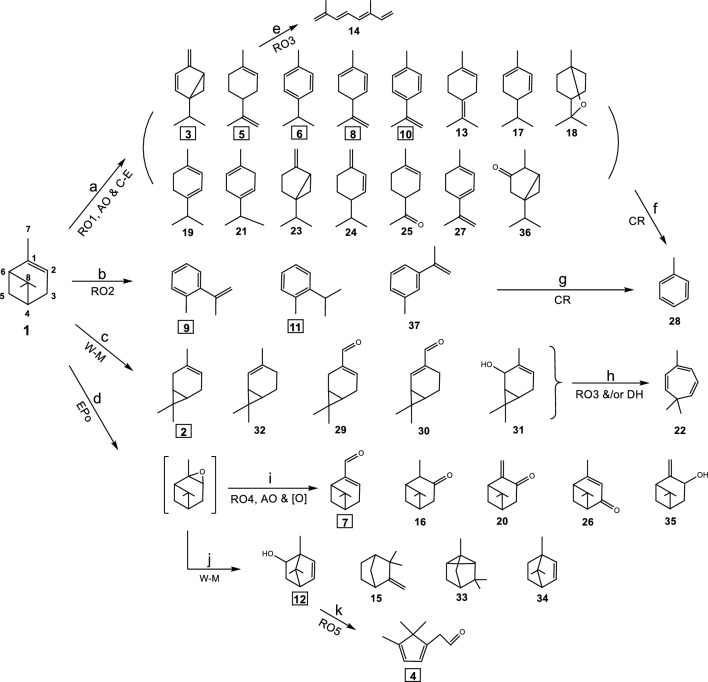
Proposed reaction pathways of α-pinene (**1**). The numbers refer to chemicals in Table 1, and boxed numbers represent the major RPs. RO: Ring opening, AO: Allylic oxidation, C-E: Cyclo-etherification reaction, W-M: Wagner-Meerwein rearrangement, EPo: Epoxidation, CR: Cleavage and removal, DH: dehydration, and [O]: Oxidation.

The oxidative reactions of α-pinene (**1**) and other monoterpenes in the gas phase have been reported to be initiated by oxidants, catalysts or rearrangement agents including ozone, hydroxyl (OH^·^) and nitrate (NO_3_^·^) radicals, hydrogen cation (acid), metal or metal compounds, as well as UV light in several chamber studies^33,36,46–48^. Figure 2 illustrates the possible reaction pathways of α-pinene for the formation of all identified RPs listed in Table 1. Ring opening (RO) of the 4-member ring in α-pinene (**1**) through cleavage of the 6,8-carbon bond (pathway a) leads to the formation of a large number of RPs (**3**, **5**, **6**, **8**, **10**, **13**, **17**, **19**, **21**, **23**, **24** and **27**, see Table 1 for chemical names). Further cleavage of the 4,5-carbon bond in limonene (**5**) (pathway e in Fig. 2) leads to the formation of cosmene (**14**). Allylic oxidation (AO) and/or cyclo-etherification reaction (C-E) of some of these RPs may be responsible for the formation of oxygen-containing RPs such as eucalyptol (**18**), limona ketone (**25**) and m-cymene (**36**). Similar cleavage of the 4,8-carbon bond to open the 4-member ring (pathway b) in α-pinene (**1**) followed by rearrangement leads to the formation of o-isopropenyltoluene (**9**), o-cymene (**11**) and m-cymene (**37**). Further reactions of some compounds from pathways a and b via cleavage and removal (CR) of the CH_3_COCH_3_ (acetone) group form toluene (**28**) (pathways f and g). Wagner-Meerwein (W-M) rearrangement of α-pinene (**1**) through migration of the 6,8-carbon bond forms either the 5,8-carbon bond (leading to 3-carene (**2**)**,** with a 4,5,7- three-member ring), or the 3,8-carbon bond resulting in 2-carene (**32**) (with a 3,4,7-three-member ring isomer) (pathway c). Further oxidation of **2** and **32** leads to the formation of 3-caren-10-al (**29**) as well as 3-caren-2-ol (**31**) and 2-caren-10-al (**30**)**,** respectively. RP 3,7,7-trimethylcyclohepta-1,3,5-triene (**22**) is likely formed from RO via cleavage of the 4,5-carbon bond (and also dehydration (DH) in some cases) of the pathway c products (especially **2**, **31** and **32**) (pathway h).

Epoxidation (EPo) of the double bond in the 6-member ring leads to the formation of α-pinene epoxide (pathway d). Although α-pinene epoxide is a well-known reaction product of α-pinene and was reported to be detected under ambient temperature, it was not detected under our thermal desorption GC/MS conditions. A likely explanation is that α-pinene epoxide may have gone through further reaction rapidly under elevated temperatures in the presence of trace moisture. Further reactions (pathway i) of α-pinene epoxide lead to the formation of myrtenal (**7**), pinocamphone (**16**), pinocarvone (**20**), levoverbenone (**26**) and pinocarveol (**35**). Through W-M rearrangement (pathway j), α-pinene epoxide generates products 1,7,7-trimethylbicyclo[2.2.1]hept-5-en-2-ol (**12**), camphene (**15**) and bornylene (**34**) (rearrangement from the 6,8-carbon bond to form the 1,8-carbon bond), as well as cyclofenchene (**33**) (rearrangement from the 6,5-carbon bond to form the 1,5-carbon bond). Cleavage of the 1,2-carbon bond in **12** further leads to the formation of α-campholenal (**4**) (pathway k).

### Reaction pathways of terpinolene

**Figure 3:**
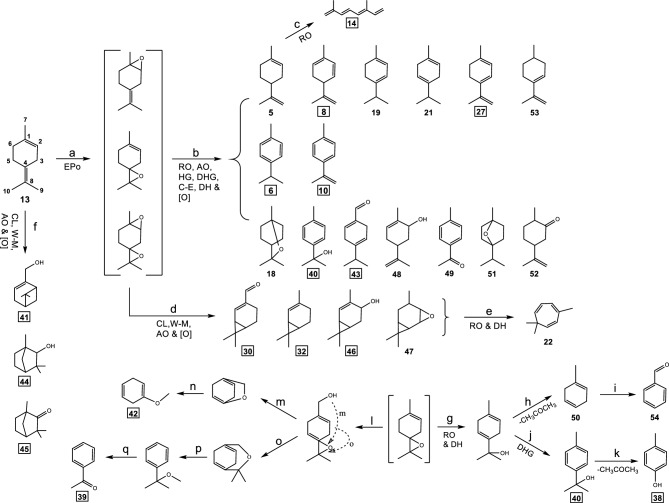
Proposed reaction pathways of terpinolene (**13**). The numbers refer to chemicals in Table 2, and boxed numbers represent the major RPs. EPo: Epoxidation, RO: Ring opening, AO: Allylic oxidation, HG: hydrogenation, DHG: Dehydrogenation, C-E: Cyclo-etherification reaction, DH: dehydration, CL: Cyclization, W-M: Wagner-Meerwein rearrangement, and [O]: Oxidation.

Based on identified RPs, reaction pathways of terpinolene (**13**) are proposed in Fig. 3. Under the experimental conditions, the double bonds in terpinolene are initially either oxidized to form epoxide at the 1,2-position or the 4,8-position or both (pathway a). These epoxides were not detected in the chromatogram as they likely further reacted rapidly with water molecules at elevated temperature, similar to α-pinene epoxide, to form diols^21^ leading to the formation of a number of RPs with the same structural skeleton as terpinolene itself but with various carbon–carbon double bonds. These RPs include limonene (**5**), p-cymene (**6**), 1,5,8-p-menthatriene (**8**), p-isopropenyltoluene (**10**), α-terpinene (**19**), γ-terpinene (**21**), 1,3,8-p-menthatriene (**27**) and p-mentha-3,8-diene (**53**) (pathway b). These RPs could also be produced through various dehydrogenation (DHG, loss of two hydrogen atoms), hydrogenation (HG, addition of two hydrogen atoms) and RO. Further RO, AO, DH, C-E and oxidation reactions of the epoxides also lead to the production of several oxygenated RPs (eucalyptol (**18**), p-cymen-8-ol (**40**), 1,4-p-menthadien-7-al (**43**), 2-methyl-5-(1-methylethenyl)-2-cyclohexen-1-ol (**48**), p-acetyltoluene (**49**), 1,4-cineole (**51**) and cis-dihydrocarvone (**52**)) (pathway b). The cyclization (CL) and W-M rearrangement of terpinolene epoxides formed a three-member ring in the structure and produced several related RPs (2-caren-10-al (**30**), 2-carene (**32**), 2-caren-4-ol (**46**) and 4,5-epoxy-carane (**47**)) after AO and hydroxylic oxidations (pathway d). Further opening of the three-member ring and DH lead to the formation of 3,7,7-trimethylcyclohepta-1,3,5-triene (**22**) (pathway e). The oxidation reaction of terpinolene also leads to the formation of the major RPs myrtenol (**41**), fenchol (**44**) and fenchone (**45**) through CL, W-M rearrangement, AO and other following oxidation reactions (pathway f).

p-Cresol (**38**) and acetophenone (**39**) had relatively high concentrations among the RPs (Table 2). Pathways of their formation are proposed as follows. Oxidative cleavage of the 4,8-double bond in terpinolene forms a 4-oxo structure that leads to the formation of **38** (pathways g, j and k) as well as several other related RPs (p-cymen-8-ol (**40**), 1-methyl-1,4-cyclohexadiene (**50**) and benzaldehyde (**54**)). Oxidization at the 7- carbon position (allyl oxidation) of the 4,8-epoxide of terpinolene followed by rearrangement leads to the formation of **39** (pathways l, o, p and q) as well as 1-methoxycyclohexa-1,4-diene (**42**) (pathways l, m and n).

### Influence of temperature and oxygen content on the formation of RPs

By mimicking the typical vaping temperature in the presence of air, which provides oxygen and trace levels of oxidants such as ozone, the impact of both temperature and amount of air on the reaction of α-pinene and terpinolene, as well as the formation of their RPs can be observed. Changing temperature and air intake altered the relative concentrations of both parent compounds and their RPs, represented by their percentage of peak areas in the GC/MS chromatogram. For the parent compounds, when temperature increased from 100 to 300 °C the percentage of α-pinene in the GC/MS chromatograms decreased from 56.94 to 41.65% (Table 1) and terpinolene from 40.76 to 31.32% (Table 2). It is interesting to observe that increased air intake also led to the decrease of unreacted α-pinene from 50.01 to 42.73% (Table 1), but had little impact on terpinolene, which remained at about 34% at three different levels of air intake (Table 2). The results probably indicate that oxidants in level-1 air intake may be sufficient in promoting the initial oxidation of terpinolene, and further increase of air intake did not have an additional effect. On the other hand, increased oxidants from level-1 to level-3 continued to enhance the effect on the initial cleavage of carbon bonds in α-pinene. The promotion effect could also be seen on the oxidations of other vaping liquid components such as PG and GLY, where an obvious decrease in oxidative reactions of PG and GLY under reduced oxygen content was reported^10^. Furthermore, no oxidation reaction was found under an inert atmosphere^49^. More interestingly, such ozone- or O_2-_initiated thermal oxidative reaction of both monoterpenes^25^ and vaping solvents (PG and GLY)^50^ could occur at significantly lower temperatures as compared to the pyrolysis under anaerobic conditions.

In this study, we noticed that temperature and air intake also influence the reaction pathways by affecting the preference of these often competing reaction pathways, leading to changes in the levels of RPs. In general, increased temperature leads to higher levels of RPs. Among the 11 major RPs of α-pinene (**1**), which had a relative concentration of 0.5% or greater, the relative concentrations of eight of them (2,4(10)-thujadiene (**3**)**,** α-campholenal (**4**), p-cymene (**6**), myrtenal (**7**), 1,5,8-p-menthatriene (**8**), o-isopropenyltoluene (**9**), p-isopropenyltoluene (**10**) and o-Cymene (**11**)) increased when temperature increased (Table 1). The amount of 3-carene (**2**) and limonene (**5**) increased between 100 and 200 °C and then decreased when the temperature continued to rise, suggesting that **2** and **5** could undergo thermal degradation at elevated temperatures. Changes in the relative concentration of 1,7,7-Trimethylbicyclo[2.2.1]hept-5-en-2-ol (**12**) were different in that the amount remained first constant between 100 and 200 °C and then increased when the temperature further increased. Compared to α-pinene (**1**), a more diverse pattern of levels among the RPs from terpinolene (**13**) was observed (Table 2). Various patterns of changes in relative concentrations with increased temperature included continuous increase (cosmene (**14**), 3,7,7-trimethylcyclohepta-1,3,5-triene (**22**), 1,3,8-p-menthatriene (**27**), fenchone (**45**) and p-acetyltoluene (**49**)), continuous decrease (p-cresol (**38**)), first increase then decrease (p-cymen-8-ol (**40**) and myrtenol (**41**)), first decrease and then increase (acetophenone (**39**)), flat first then increase (p-cymene (**6**) and 1,4-cineole (**51**)), decrease first and then flat (eucalyptol (**18**), 1-methyl-1,4-cyclohexadiene (**50**) and p-mentha-3,8-diene (**53**)), increase first and then flat (α-terpinene (**19**), fenchol (**44**) and 2-caren-4-ol (**46**)), and constant (1,5,8-p-menthatriene (**8**) and 2-carene (**32**)).

With increased air intake the amount of 2,4(10)-Thujadiene (**3**) formed from α-pinene (**1**) continuously increased, while the amount of 3-carene (**2**) continuously decreased (Table 1). The amount of α-campholenal (**4**) however, increased first from level-1 to level-2 then decreased from level-2 to level-3, while the amount of limonene (**5**) decreased from level-1 to level-2 and then remained nearly the same from level-2 to level-3. On the other hand, the amount of p-cymene (**6**) remained unchanged. These different variations in the levels of RPs demonstrated that the oxidative thermal reaction processes of α-pinene are complex including both parallel and consecutive reaction pathways.

Although little influence of air intake on the levels of terpinolene (**13**) was observed, oxygen does show an effect on the subsequent reactions of the primary RPs and the transformation reactions among them, resulting in changes in the levels of RPs (Table 2). For example, when air intake levels increased, the amount of p-isopropenyltoluene (**10**) and 2-caren-4-ol (**46**) first increased then decreased, while p-cymene (**6**), 1,3,8-p-Menthatriene (**27**) and 1,4-p-Menthadien-7-al (**43**) showed a continuous increase and acetophenone (**39**), p-cymen-8-ol (**40**), fenchol (**44**) and 4,5-epoxy-carane (**47**) continuously decreased, (Table 2). This indicates that while varying oxygen content had little effect on the initial reaction of terpinolene, it influenced subsequent oxidation reactions leading to the formation of RPs. In addition, we also found that change in air intake levels could completely change some reaction pathways in the oxidative thermal reaction of terpinolene (**13**) resulting in the formation of specific RPs. For example, RPs myrtenol (**41**) and cosmene (**14**) were detected only at air intake level-1, while 1-methoxycyclohexa-1,4-diene (**42**) and 1,4-p-menthadien-7-al (**43**) only at level-2 and level-3 (Table 2), indicating the preferred reaction pathways for the production of **41** and **14** at lower O_2_ level was not favorable for the production of **42** and **43**, and vice versa.

The presence of multiple parallel and consecutive reaction pathways in the formation of many RPs indicates that temperature can also influence the reaction pathways. For example, levels of several RPs (e.g., 2-caren-10-al (**30**) and p-cresol (**38**)) of terpinolene (**13**) decrease with increasing temperature (columns 10–12 in Table 2). However, the decrease of these products may not indicate negative promotion of temperature. It could be that one or more competitive parallel may be more favorable or the RPs become more reactive at higher temperatures. For some products, partial complete reaction into methane (CH_4_), ethylene (C_2_H_4_) and H_2_ may also occur at higher temperatures^51^. This is another possible reason for the decrease of levels of these RPs at higher temperatures.

### Implications for exposure

Thermal reaction of monoterpenes, including α-pinene, can occur over a wide temperature range. However, low-temperature oxidative reaction has gained increasing attention due to its relevance to the reaction processes in vaping^21^. A study conducted in a low-temperature smog chamber^33^, which involved heating α-pinene at temperatures between 90 and 130 °C, showed that approximately 23% to 37% of α-pinene underwent rearrangement or oxidation in the presence of air.

Temperature to thermally extract vaping liquid components is controlled through either temperature setting or voltage setting. For example, the lowest and highest temperature settings in common temperature controlled device can result in a heating coil temperature of around 420 °C and 246 °C, while voltage controlled devices at settings of 4.0 V and 3.2 V result in 543 °C and 450 °C, respectively^52^. Our study demonstrated that thermal reactions can occur at much lower temperature range. Formation of a large number of RPs with considerably high levels at such low temperature implies that inhaled vapor during vaping not only contains evaporated components from vaping liquids but also many RPs of these components. Despite the popularity of vaping^1–4^ and quick market expansion of vaping products^5^, the exposures and health risks related to vaping are not fully understood. By showing the complex nature of both reaction products and reaction pathways under conditions milder than those in vaping, we have clearly demonstrated that consumers who use vaping products could be exposed to both vaporised vaping liquid components and their reaction products formed during vaping.

## Conclusions

Many thermal reaction products of α-pinene and terpinolene were putatively identified in this study. Possible pathways were proposed to explain the formation of these reaction products. Both reaction temperature and air intake in the reaction mixture have shown various effects on the levels of parent chemicals as well as their reaction products, indicating a competitive nature of thermal reaction pathways under conditions that mimic the vaping practice.

Vaping liquid is a mixture of water, food grade flavoring agents^1–3^, a choice of nicotine or cannabis levels, and carrier substances such as PG or vegetable glycerin (VG)^10,11^. The results presented in this paper are based on thermal reactions of single chemicals. In real-world, thermal reactions occur in the presence of mixture of chemicals in vaping liquids. A more complex formation profile of RPs is expected for the reactions of the mixture of chemicals. Identification of RPs from single chemicals and knowledge on the formation of these RPs can help inform research in this area. Our future research will focus on investigating other thermally liable compounds used in vaping liquids to gain more knowledge on the possible human exposure to chemicals both present in vaping liquids and generated during vaping.

## Limitations

There are several limitations in this study. Firstly, there is the lack of confirmation for the reaction products using authentic standards. Instead, we rely on NIST mass spectrum library for the identification. Lack of structure confirmation may introduce some uncertainties in the identification of reaction products. Secondly, the relative concentrations of compounds detected in the samples were estimated using percentage of peak areas by assuming the same response factor for all compounds, which likely are not the case. As a result, discussions on the influence of thermal desorption temperature and air intake amount should be interpreted with caution. Lastly, despite our best efforts, the in-situ thermal desorption GC/MS setup does not equal to continuous puffing/heating/cooling cycles in real-world vaping and glass wool deposited with test chemical does not equal to the metal surface of the heating coil in vaping devices. These limitations may introduce uncertainties when the results are used for assessing human exposure and should be addressed in future research.

### Supplementary Information

Supplementary Information.

## Data Availability

All data generated or analysed during this study are included in this published article. Original GC/MS datafiles of this study are available from the corresponding author on reasonable request.
