# Author Correction: Thermal reaction products and formation pathways of two monoterpenes under in situ thermal desorption conditions that mimic vaping coil temperatures

**DOI:** 10.1038/s41598-024-54633-5

**Published:** 2024-03-04

**Authors:** Jianjun Niu, Jiping Zhu

**Affiliations:** Exposure and Biomonitoring Division, Environmental Health Science and Research Bureau, Health Canada, Ottawa, Canada

Correction to: *Scientific Reports* 10.1038/s41598-023-49174-2, published online 08 December 2023

The original version of this article contained errors in Table 1 and the captions of Table 1 and 2.

In the original version of this Article, Table 1 was incorrectly indexed.

Table 1

**Table:** 

C1ompound number^a^	Compound name	Formula
**1**	α-Pinene	C_10_H_16_

now reads

Table 1

**Table:** 

Compound number^a^	Compound name	Formula
1	α-Pinene	C_10_H_16_

In addition, in the Legend of Table 1, where

“Identified reaction products of α-pinene that have percent area of 0.01% or greater. ^a ^Boxed number represents a major reaction product (RP); ∑Unknown: Total unknown product peaks.”

now reads

“Identified reaction products of α-pinene that have a percent area of 0.01% or greater. ^a ^Bold number represents a major reaction product (RP); ∑Unknown: Total unknown product peaks.”

Furthermore, in the Legend of Table 2, where

“Identified reaction products of terpinolene that have percent area of 0.01% or greater. ^a ^Boxed number represents a major reaction product (RP); ∑Unknown: Total unknown product peaks.”

now reads

“Identified reaction products of terpinolene that have a percent area of 0.01% or greater. ^a ^Bold number represents a major reaction product (RP); ∑Unknown: Total unknown product peaks.”

The original Article has been corrected.

