# Supplementary material for: Thermal reaction products and formation pathways of two monoterpenes under in situ thermal desorption conditions that mimic vaping coil temperatures
Source: Sci Rep. 2023 Dec 8;13:21650. doi: 10.1038/s41598-023-49174-2 (PMC10709557; doi:10.1038/s41598-023-49174-2)
Supplement: Supplementary file 1 — Supplementary Information. [file 41598_2023_49174_MOESM1_ESM.pdf]

Supplementary Material

Thermal Reaction Products and Formation Pathways of Two Monoterpenes under *In Situ* Thermal Desorption Conditions That Mimic Vaping Coil Temperatures

Jianjun Niu and Jiping Zhu*

Exposure and Biomonitoring Division, Environmental Health Science and Research Bureau, Health Canada, Ottawa, Canada

Each of the two monoterpenes (α-pinene and terpinolene) were tested three different air intake levels at constant temperature of 200 °C and at three different temperatures (100 °C, 200 °C and 300 °C) at constant air intake level-1. This file contains four figures (S1 to S4) showing thermal desorption GC/MS chromatograms of these experiments.

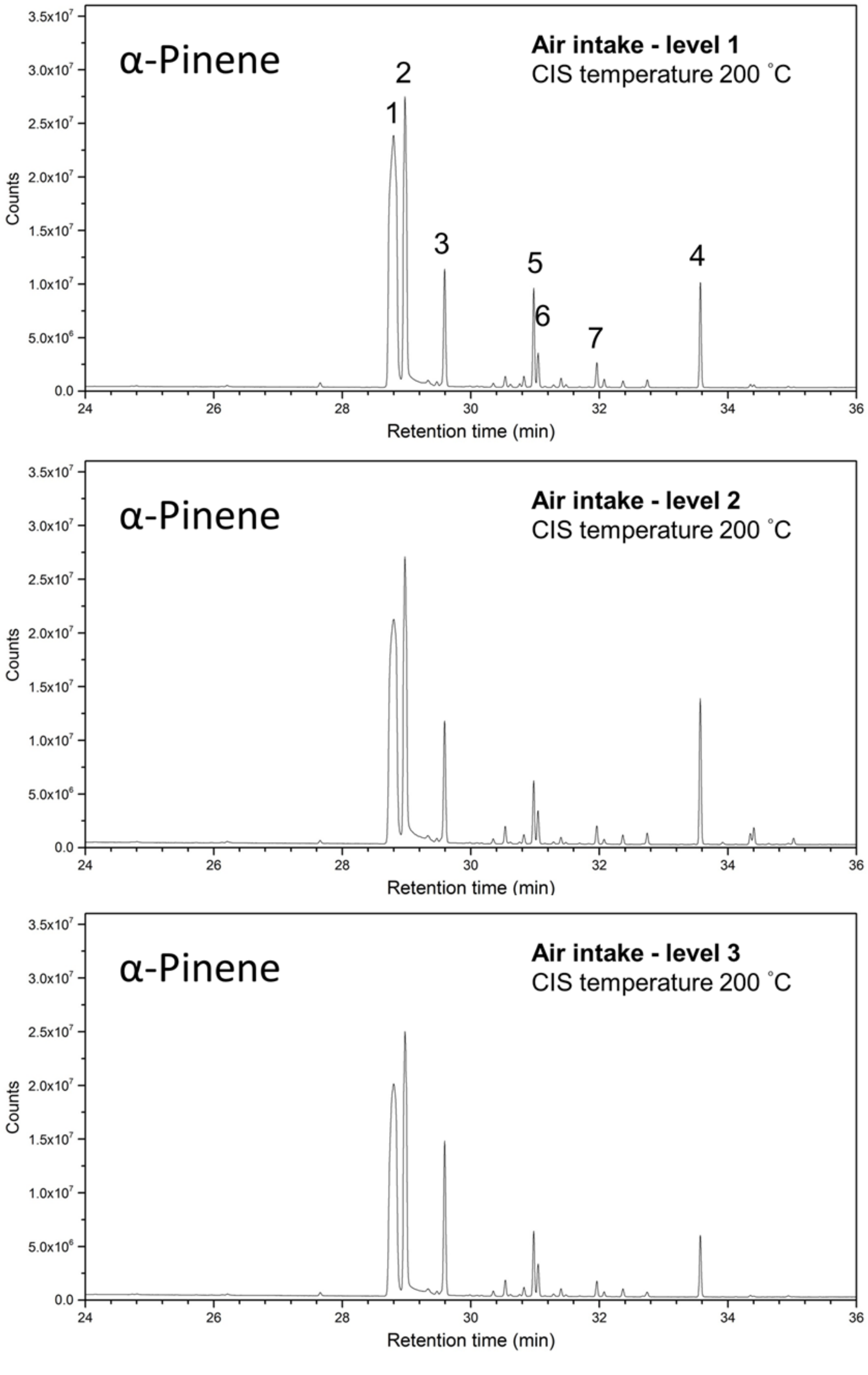

Figure S1: Thermal desorption GC/MS chromatograms of α-pinene: Effects of air intake level

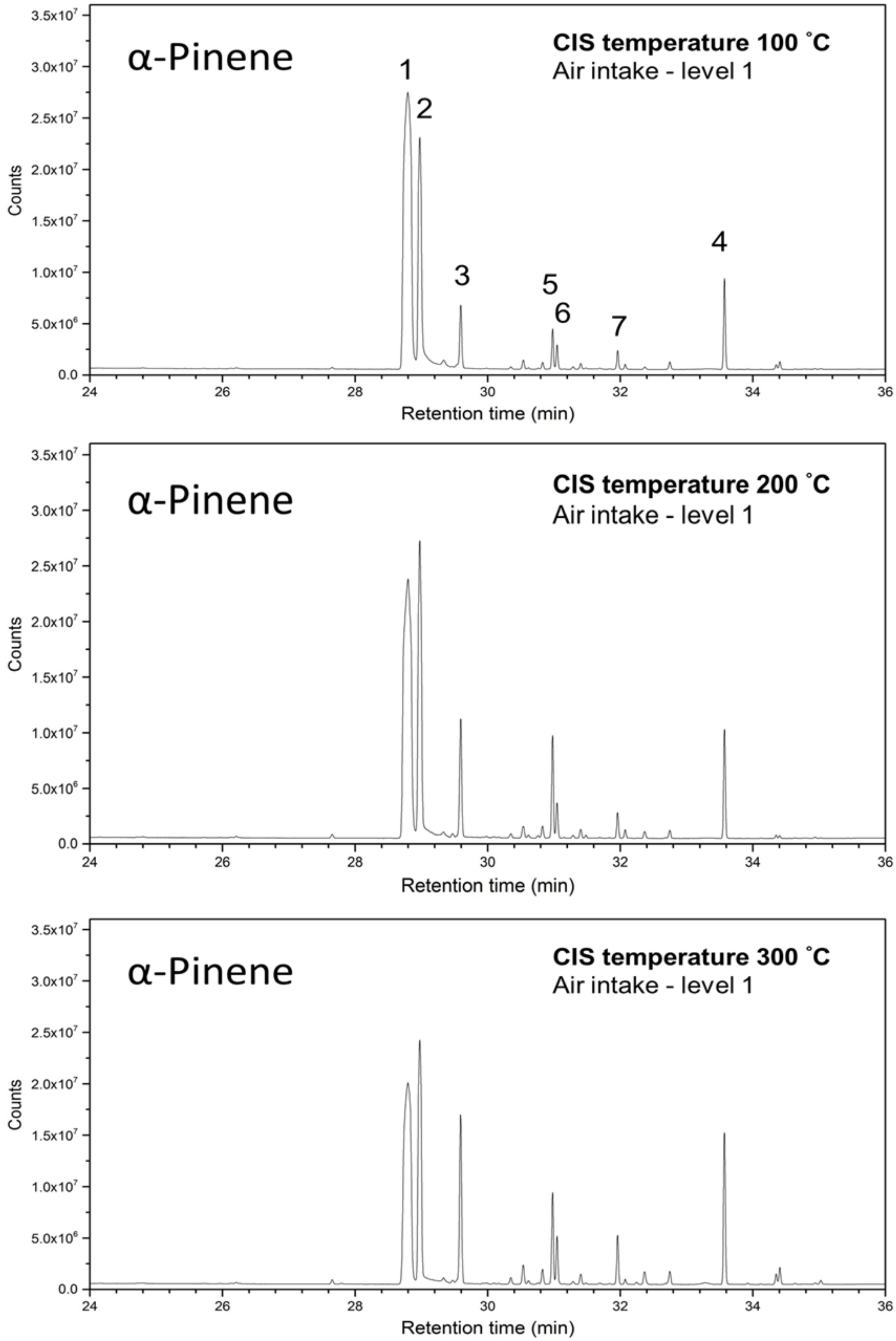

Figure S2: Thermal desorption GC/MS chromatograms of α-pinene: Effects of temperature

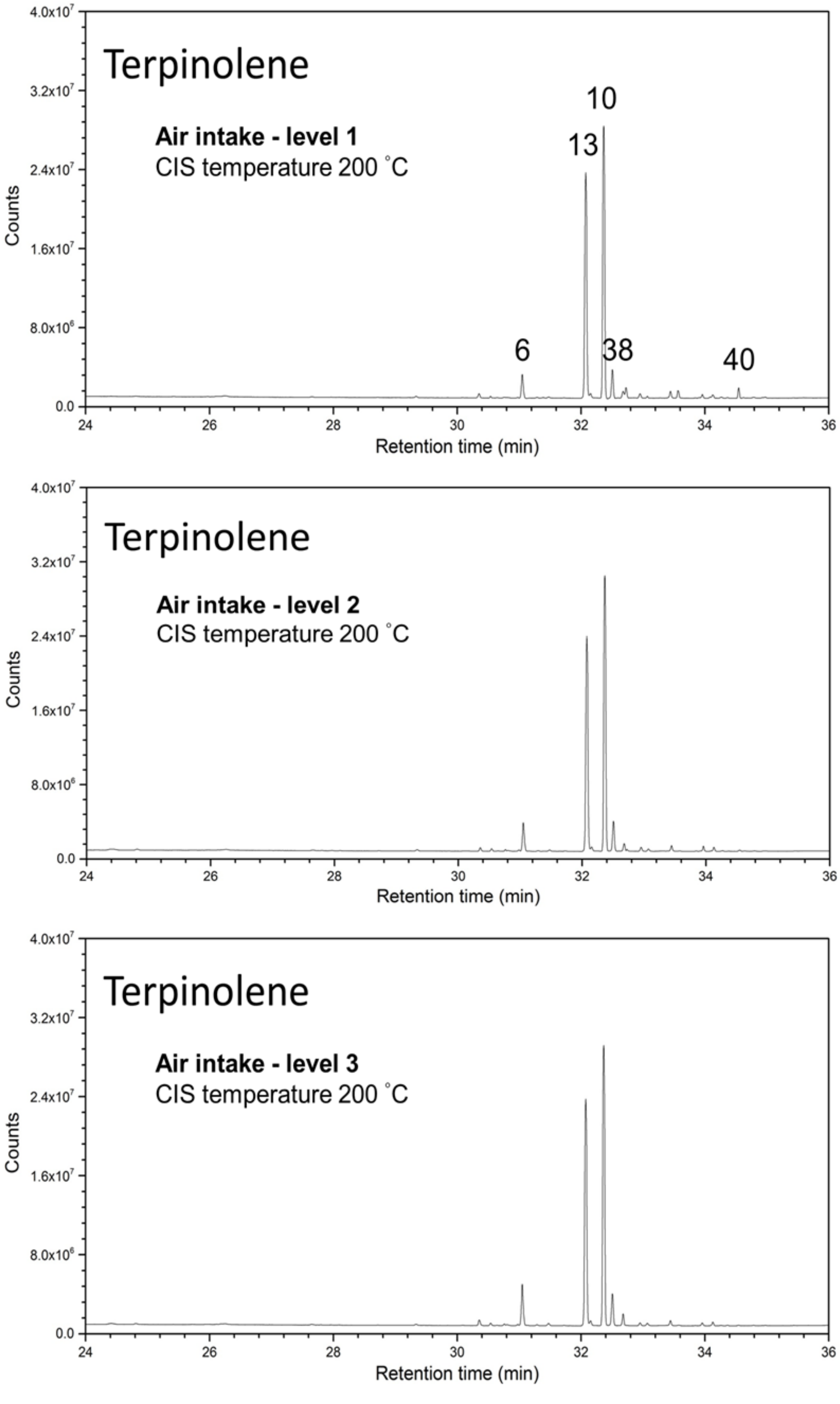

Figure S3: Thermal desorption GC/MS chromatograms of terpinolene: Effects of air intake level

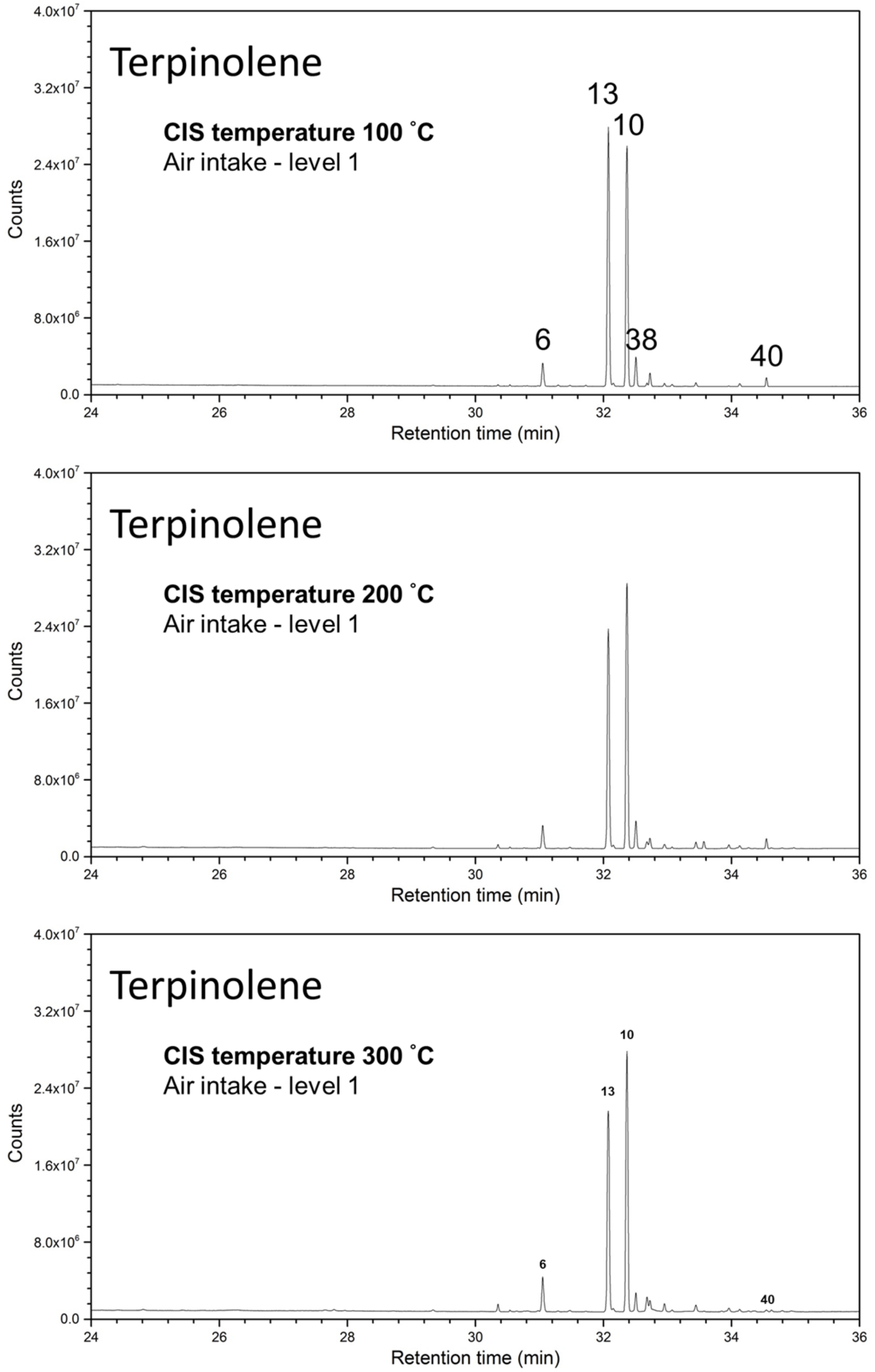

Figure S4: Thermal desorption GC/MS chromatograms of terpinolene: Effects of temperature
